# The Pleiotropic Influence of Cannabidiol and Tetrahydrocannabinol on Inflammatory Biomarkers: A Systematic Review and Meta-Analytical Synthesis

**DOI:** 10.3390/ijms262311618

**Published:** 2025-11-30

**Authors:** Bruno Moreira Candeloro, Camila M. de Oliveira, Fabiana Veronez Martelato Gimenez, Marianne P. C. N. Barbosa, Beatriz Paiva Soares, Ana C. F. Ruiz, Derfel R. M. A. Folegatti, Sandra Maria Barbalho, Nancy S. Oliveira, Andrey A. Porto, David Matthew Garner, Fernando H. Sousa, Vitor E. Valenti

**Affiliations:** 1 Systematic Reviews Center for Cardiovascular and Metabolic Health, School of Philosophy and Sciences, São Paulo State University, Marília 17525-900, SP, Brazil; bruno.candeloro@unesp.br (B.M.C.); fabiveronez@hotmail.com (F.V.M.G.); marianne.penachini@unesp.br (M.P.C.N.B.); beatriz.p.soares@unesp.br (B.P.S.); clara.franchi@unesp.br (A.C.F.R.); derfel.folegatti@unesp.br (D.R.M.A.F.); nancy.oliveira@unesp.br (N.S.O.); aa.porto@unesp.br (A.A.P.); drfhs@yahoo.com.br (F.H.S.); 2 Department of Biochemistry and Pharmacology, School of Medicine, Universidade de Marília (UNIMAR), Marília 17525-902, SP, Brazil; marcondes.oliveira@unesp.br; 3 Professor of the Nursing Course and the Postgraduate Program in Health and Aging, Faculdade de Medicina de Marília (FAMEMA), Marília 17519-030, SP, Brazil; 4 Postgraduate Program in Structural and Functional Interactions in Rehabilitation, School of Medicine, Universidade de Marília (UNIMAR), Marília 17525-902, SP, Brazil; 5 Research Coordinator, UNIMAR Charity Hospital, Universidade de Marília (UNIMAR), Marília 17525-902, SP, Brazil; 6 Department of Biochemistry and Nutrition, School of Food and Technology of Marília (FATEC), Marília 17500-000, SP, Brazil; 7 Cardiorespiratory Research Group, Faculty of Health and Life Sciences, School of Biological and Medical Sciences, Oxford Brookes University, Headington Campus, Gipsy Lane, Oxford OX3 0BP, UK; p0039985@brookes.ac.uk

**Keywords:** cannabidiol (CBD), Δ9-tetrahydrocannabinol (THC), inflammatory biomarkers, systemic inflammation, systematic review and meta-analysis

## Abstract

Preclinical data suggest that cannabidiol (CBD) and Δ9-tetrahydrocannabinol (THC) modulate inflammatory pathways (e.g., NLRP3, NF-κB, and PPAR-γ), but clinical translation into consistent changes in circulating biomarkers remains ambiguous. Two reviewers independently screened the studies, extracted data, and assessed risk of bias with RoB-2. Random-effects meta-analyses (RevMan 5.4.1) formed standardized mean differences (SMD) or mean differences (MD) as appropriate. The certainty of evidence was graded by means of GRADE. Thirteen studies satisfied inclusion criteria; meta-analyses were feasible for IL-6 (four studies, n ≈ 129 per arm), IL-8 (two studies, n ≈ 78 per arm), IL-10 (two studies, n ≈ 92 per arm), and TNF-α (three studies, n ≈ 105 per arm). Pooled estimates favored CBD but were trivial and imprecise: IL-6 SMD −0.17 (95% CI −0.56 to 0.23; *p* = 0.41; I^2^ = 55%); IL-8 SMD −0.30 (95% CI −0.62 to 0.01; *p* = 0.06; I^2^ = 0%); IL-10 SMD −0.10 (95% CI −0.83 to 0.63; *p* = 0.79; I^2^ = 81%); and TNF-α SMD −0.09 (95% CI −0.45 to 0.27; *p* = 0.62; I^2^ = 33%). Individual trials reported reductions in biomarkers in high-exposure or diseased populations. GRADE ratings were as follows: IL-6 very low, IL-8 moderate, IL-10 low, and TNF-α moderate. Current RCT evidence demonstrates inconsistent, often trivial effects of phytocannabinoid interventions on circulating inflammatory biomarkers.

## 1. Introduction

Non-pharmacological and pharmacological therapies have gained increasing attention for their ability to modulate inflammatory markers and cardiovascular parameters [1,2,3]. With this in mind, cannabinoids such as cannabidiol (CBD) and Δ9-tetrahydrocannabinol (THC) have garnered substantial scientific and clinical interest for their alleged immunomodulatory properties. Preclinical studies emphasize CB2 receptor signaling and associated microglial repolarization, mainly in neuro-centered reviews [4]. Mechanistic research—chiefly fixated on CBD—describes the inhibition of NF-κB, suppression of NLRP3 inflammasome assembly, and activation of PPAR-γ, leading to cell-type-specific reductions in pro-inflammatory mediators [5]. Together, these pathways provide biological plausibility for the anti-inflammatory effects of phytocannabinoids across neurological, cardiometabolic, and dermatological systems.

Nevertheless, translating promising preclinical signals into consistent changes in systemic inflammatory biomarkers has been limited. Randomized controlled trials and observational studies account for the mixed results with typical markers (e.g., IL-6, TNF-α, IL-1β, and CRP), and many clinical studies are limited by small sample sizes, heterogeneous populations, and variable timing of biomarker assessment. For instance, exploratory RCTs evaluating multi-week CBD administration have reported modest, variable, or null changes in circulating markers, despite advances in some clinical or surrogate outcomes (e.g., reductions in IL-6) [6]). Systematic reviews of clinical CBD trials similarly highlight a limited and heterogeneous evidence base, leading to uncertainty in pooled conclusions [7].

Several procedural and intervention-related factors likely contribute to this heterogeneity. First, formulation and route of administration (oral isolates, full-spectrum extracts, inhaled products, topical and nano-formulated preparations) markedly affect bioavailability and tissue exposure: Topical and nano-enabled systems can attain high local concentrations with slight systemic spillover, producing distinct local biomarker changes that may be unnoticed in peripheral cytokine panels [8,9]. Second, dose, treatment duration, and the target population (healthy volunteers, psychiatric or substance-use cohorts, patients with inflammatory disease) modify both the magnitude and direction of biomarker responses. Third, variability in laboratory assays, sampling windows, and outcome selection (single cytokine versus composite inflammatory profiles) further impedes direct comparison across trials [10]. These factors jointly support pre-specified subgroup analyses by formulation, route, dose, and population in future syntheses, as well as the inclusion of PK/PD endpoints (systemic and, where viable, tissue concentrations) to link exposure with biomarker changes [8,9].

A focused, biomarker-centered quantitative synthesis is thus opportune. A meta-analysis can (1) estimate pooled effects across controlled studies; (2) examine heterogeneity attributable to route, formulation, dose, and population by subgroup analyses or meta-regression; and, finally, (3) assess credibility via standard risk-of-bias and certainty-of-evidence frameworks.

Methodological transparency is vital; structured tools, such as the Cochrane RoB-2 instrument and GRADE, allow for a standardized assessment of bias, inconsistency, imprecision, and indirectness, producing evidence profiles that are informative for clinicians and trialists [11].

From a translational perspective, distinguishing between local (tissue) and systemic immunomodulation is critical. Preclinical work often reports pathway-specific effects, for example, the suppression of NLRP3 assembly and downstream IL-1β release by CBD in microglia (observed in LPS-challenged BV2 cells) [12]. Still, such tissue-level outcomes do not always translate into measurable changes in peripheral blood after systemic dosing. This incongruity is attributable to compartmental pharmacokinetics, exposure differences, and study design. It accentuates the necessity to specify the biological compartment of interest (serum/plasma versus tissue type) and to pair PD sampling with PK measurements in clinical studies [13].

Clinically, clarifying the impact of phytocannabinoids on inflammatory biomarkers has two key implications. If consistent systemic anti-inflammatory effects are established in rigorous RCTs, phytocannabinoids could inform adjunctive strategies for conditions where cytokine dysregulation is causal. Similarly, if its effects are primarily local or formulation-dependent, clinical use should prioritize route-matched indications (e.g., topical CBD for dermatological conditions). Systematic reviews of human pharmacokinetics highlighted marked erraticism in exposure by route and formulation, reinforcing the requirement for PK/PD-driven trial design [14]. At the same time, clinical PK/PD sub-studies reveal substantial interindividual variability and practical sampling challenges, which are relevant to endpoint selection and power calculations [15].

Taken together, the context-dependent and compartment-specific nature of phytocannabinoid effects imposes a systematic, quantitative assessment of clinical evidence [16]. For instance, a randomized crossover study in well-trained athletes found no change in IL-6 or IL-10 after short-term oral CBD, illustrating how population, dose, and timing can yield null results in otherwise mechanistically plausible interventions [17]. This systematic review and meta-analysis aims to quantify the effect of CBD- and THC-containing interventions on circulating inflammatory biomarkers (e.g., primary examples: IL-6, TNF-α, IL-1β, and CRP), study key effect modifiers (e.g., route, formulation, dose, duration, and population), weigh evidence certainty, and provide realistic recommendations for forthcoming clinical trials and clinical practice.

## 2. Materials and Methods

### 2.1. Protocol and Registration

This review was reported to be consistent with the Preferred Reporting Items for Systematic Reviews and Meta-Analyses (PRISMA) statement [18] and is listed in PROSPERO (CRD420250651408).

### 2.2. Eligibility Criteria

We included studies published in the database from database inception through April 2025. Eligibility followed PICOS (Population, Intervention, Comparison, Outcomes, Study design):

1. **Study design (S).** Non-randomized, randomized controlled trials (parallel or crossover, single- or double-blind) were suitable for this study. We likewise screened postgraduate theses and full-text articles from peer-reviewed scientific journals. Conference abstracts, case reports, descriptive studies, editorials, and reviews were omitted.
2. **Intervention (I).** Administration of cannabidiol (CBD), Δ9-tetrahydrocannabinol (THC), or combinations thereof, by any route or formulation (e.g., oral, inhaled, sublingual, topical, or nanoformulation). Studies evaluating other cannabinoids (e.g., CBG and CBN) without CBD/THC, or synthetic cannabinoids not classified as phytocannabinoids, were omitted. In line with this criterion, lenabasum, being a synthetic cannabinoid rather than a phytocannabinoid, was reconsidered for exclusion, as its inclusion would violate the predefined eligibility framework. Although the majority of the included clinical trials investigated isolated cannabidiol, THC was retained in the eligibility criteria because both compounds share overlapping mechanistic pathways, particularly in modulating cytokine signaling via CB1/CB2 receptors, and because preclinical evidence indicates that their anti-inflammatory effects may be synergistic or divergent [19]. Including both cannabinoids allowed us to capture the full scope of phytocannabinoid-based interventions with potential immunomodulatory effects, avoid an unduly narrow search strategy, and reduce selection bias in accordance with systematic review methodological standards.
3. **Comparator (C).** Placebo, no intervention, or an active anti-inflammatory comparator. Studies lacking a clearly defined control group were omitted.
4. **Outcomes (O).** Quantitative measures of inflammatory biomarkers, including systemic biomarkers (e.g., circulating C-reactive protein [CRP]; serum or plasma interleukins such as IL-6, IL-1β, IL-10 and TNF-α) and tissue-specific biomarkers (e.g., cytokine expression assessed in localized tissue samples or biopsy-derived assays). Studies that did not report quantitative biomarker data in either systemic or tissue-specific form were omitted.
5. **Study design (S).** Randomized controlled trials (parallel or crossover, single- or double-blind) were suitable for this study. We likewise screened postgraduate theses and full-text articles from peer-reviewed scientific journals. Conference abstracts, case reports, descriptive studies, editorials, and reviews were omitted.

### 2.3. Information Sources, Search Strategy, and Study Selection

We searched LILACS, MEDLINE/PubMed (NLM), Scopus, and Web of Science from database inception up until April 2025. The entire search strategy and exact search strings are provided in Table 1. In addition, to minimize publication bias, we systematically screened gray literature sources and major clinical trial registries, including ClinicalTrials.gov, the WHO International Clinical Trials Registry Platform, and relevant conference proceedings, to identify unpublished or ongoing studies. Retrieved records were transferred to Rayyan QCRI for deduplication and screening. Titles and abstracts were screened autonomously by two reviewers; full texts were assessed independently by two reviewers. Disagreements were resolved by discussion and, where needed, by consulting a third reviewer. After final study selection, the review team assessed the practicability of the meta-analysis.

### 2.4. Data Collection and Extraction

Two reviewers independently extracted data via a standardized form. Extracted items involved study identifiers (authors, year), design, participant characteristics, intervention and comparator details (e.g., formulation, route, dose, and duration), timing of biomarker sampling, outcomes (means and dispersion measures), and funding sources. For graphical data, we used WebPlotDigitizer^®^ to extract numerical values when authors did not provide tabulated results. Corresponding authors were contacted to request missing data. Values reported as standard errors (SEs) or confidence intervals (CIs) were converted to standard deviations (SDs) using established methods.

### 2.5. Data Items

We extracted the pre-specified inflammatory biomarker measures and, where possible, collected both pre- and post-intervention values, as well as between-group comparisons. We similarly extracted participant and intervention characteristics to strengthen subgroup analyses. Outcomes for which required data could not be obtained were included in the narrative synthesis but omitted from quantitative pooling.

### 2.6. Risk of Bias Assessment

The risk of bias was assessed using the Cochrane RoB-2 tool [11]. The five domains considered were randomization process, deviations from intended interventions, missing outcome data, measurement of the outcome, and selection of the reported result. Each domain and the overall bias judgment were rated as ‘low risk’, ‘some concerns’, or ‘high risk’. Two reviewers independently finalized RoB-2 assessments; a third reviewer arbitrated disagreements. Reviewers completed calibration training before the evaluation.

### 2.7. Certainty of Evidence

We assessed the certainty of evidence using the GRADE approach, as implemented in GRADEpro GDT v4. For each primary outcome, we considered study limitations, inconsistency, imprecision, indirectness, and publication bias to assign a certainty rating (high, moderate, low, or very low).

### 2.8. Qualitative Synthesis

We achieved a narrative synthesis that describes study characteristics, interventions, biomarker assays, and main findings. The main study details and results were tabulated. When quantitative pooling was not possible, findings were summarized narratively and by outcome domain.

### 2.9. Quantitative Synthesis and Summary Measures

Feasibility for meta-analysis was calculated after study selection. When pooling was likely, we extracted post-intervention group means and SDs (or change scores with their SDs) and computed effect sizes. We used the mean difference (MD) when outcomes were measured in the same units, or the standardized mean difference (SMD) when measures varied across studies. Where SDs were missing, we either considered them or imputed them from SEs, CIs, *p*-values, or other reported statistics, following Cochrane guidance.

Heterogeneity was assessed using the I^2^ statistic and interpreted according to Cochrane thresholds (0–29% may not be important; 30–49% may represent moderate heterogeneity; 50–74% may be substantial; 75–100% may be considerable). Statistical significance for pooled effects was set at *p* < 0.05. Random-effects models were enforced for all meta-analyses to account for between-study variability. Results were conveyed with effect estimates (MD or SMD), 95% CIs, and *p*-values, and exhibited in forest plots. All analyses were led in Review Manager (RevMan 5.4.1).

## 3. Results

### 3.1. Study Selection

**Figure 1:**
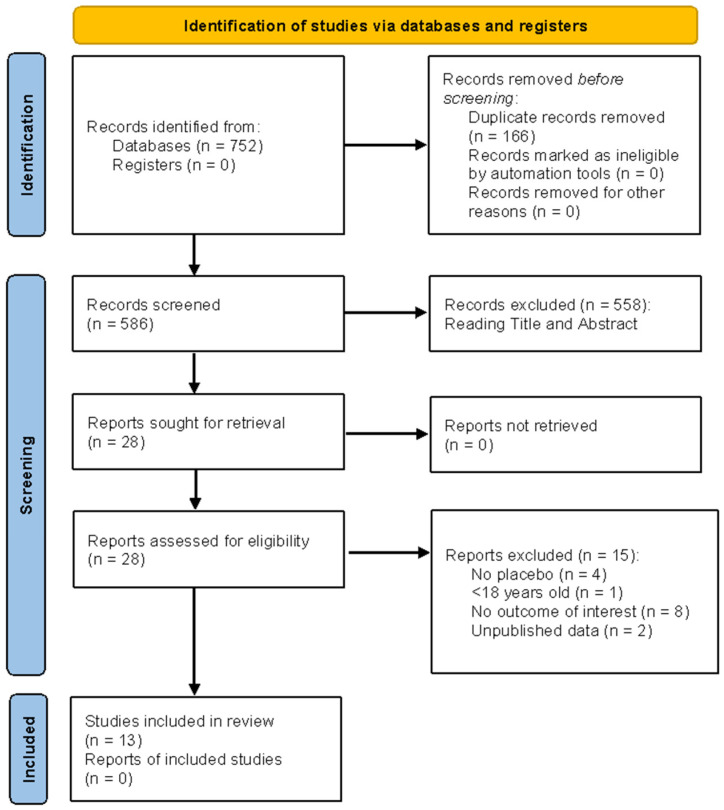
PRISMA flow chart of the literature search process.

Figure 1 illustrates the study-selection process for this review. A total of 752 records were identified through database searches; no records were retrieved from registers. After eliminating 166 duplicates, 586 records were screened by title and abstract (data Supplementary Materials). Next, 558 records were omitted, and 28 full-text reports were retrieved and assessed for eligibility. Fifteen reports were excluded at full text for the following reasons: no placebo used (n = 4); participants under 18 years (n = 1); outcomes not of interest (n = 8); unpublished data (n = 2). Thirteen studies satisfied eligibility criteria and were included in the final systematic review.

### 3.2. Study Results

Table 2 summarizes the characteristics and results of the studies.

Cohen et al., 2023 [20] topical CBD + eicosapentaenoic acid (EPA): in vitro HaCaT assays demonstrated reduced UVB-induced IL-8 and PGE2 production with CBD, an effect potentiated by EPA. An ex vivo human skin model indicated (~40%) restoration of epidermal viability after UVB damage and improved extracellular-matrix histology. In a 56-day clinical trial (n = 33 females, 45–65 years), the design produced significant improvements in wrinkle area/volume, an 8.8% decrease in sub-epidermal low-echogenicity band thickness, a 25.6% increase in elasticity, a 31.2% increase in hydration, and a 15% reduction in red spots. The participant satisfaction was high.

Flores et al. (2023) [21] oral CBD 50 mg/day for 8 weeks in healthy, physically active adults: No significant between-group differences for body composition, aerobic fitness, muscle strength, physical activity, cognitive measures, psychological well-being, or resting CRP were observed. The placebo group experienced reductions in peak anaerobic power (~9.6%) and relative peak power (~6.6%) that were undetected in the CBD group; mental-health scores declined slightly in both groups (pandemic context noted).

Gao et al. (2022) [22] JW-100 topical formulation (CBD + aspartame) for atopic dermatitis (n = 66): after 14 days, JW-100 produced a greater mean reduction in ISGA score (−1.28) than isolated CBD (−0.81) or placebo (−0.71). Fifty percent of JW-100 patients achieved an ISGA score of 0–1, compared with 20% (CBD) and 15% (placebo). No adverse events were reported.

Gurgenci et al. (2023) [23] reported that, in advanced cancer patients, isolated CBD produced no significant change in CRP over time compared with placebo. A cytokine sub-study (n = 33) detected no group differences in trajectories of inflammatory markers. The associated chemotherapy did not alter the results.

Isenmann et al. (2024) [17] evaluated short-term oral CBD in athletes with training-induced muscle damage (increased creatine kinase, myoglobin) after intensive training. CBD reduced the increase in myoglobin in advanced athletes but had no consistent effect on performance decline, inflammatory markers (IL-6, IL-10), or oxidative stress indicators. A slight inhibitory trend in the platelet-to-lymphocyte ratio was observed in some advanced athletes.

Jirasek et al. (2024) [24] evaluated, in a preclinical and clinical periodontal study, the in vitro effects of CBD on IL-6 and IL-8 in human gingival fibroblasts challenged with *Porphyromonas gingivalis*, demonstrating antimicrobial activity. In a 56-day clinical trial (n = 90), CBD improved periodontal indices (Gingival Index, Gingival Bleeding Index, Modified Gingival Index) compared with the placebo, with efficacy equivalent to that of chlorhexidine. No adverse effects were reported.

Johnson et al. (2025) [25] CBD 298 mg and exercise in heat: no significant effect on core or skin temperature, heart rate, sweat loss, subjective responses, or intestinal-damage marker (I-FABP) was observed versus placebo. A mild anti-inflammatory signal was proposed: plasma CD14 was lower 90 min post exercise with CBD, and IL-6 achieved a discrete reduction.

Mastrofini et al. (2024) [26] hemp-derived CBD in healthy adults: no significant differences in inflammatory/immunological biomarkers, perceived stress, sleep quality, or mood were observed. The CBD group experienced lower pain (as measured by the Fundamental Pain Index) compared to the placebo group, and the supplement was well-tolerated.

Morissette et al. (2021) [6] high-dose CBD (800 mg/day, 92 days) in subjects with a cocaine-use disorder: The CBD group presented with significantly lower IL-6 and VEGF-A versus placebo, as well as improved CD25^+^CD4^+^ regulatory T cells; no changes were detected in total B cells, CD4^+^ T cells, or CD8^+^ T cells.

Sahinovic et al. (2022) [27] single 300 mg CBD dose in trained endurance athletes: throughout submaximal exercise, CBD increased VO_2_ at some points and subjective pleasure; during incremental exercise, CBD increased VO_2max_ and RER_max_ but did not affect time to exhaustion. Post-exercise, CBD may have reduced IL-1β but increased myoglobin.

Spiera et al. (2020) [28] lenabasum (synthetic cannabinoid receptor agonist) in diffuse cutaneous systemic sclerosis: Safety was found to be equal to placebo; lenabasum improved CRISS, MRSS, patient-reported symptoms, and itch scores versus the placebo.

Urlic et al. (2023) [29] primary hypertension: five weeks of CBD reduced serum IL-8, IL-10, and IL-18 from baseline in the CBD group but not in the placebo group; reductions in IL-8 and IL-10 correlated positively with diastolic-BP reduction.

Utomo et al. (2017) [30] ex vivo human PBMCs: medicinal cannabinoids shaped immunosuppression by means of inhibition of p38 MAPK, mTOR, and ERK pathways. THC pre-treatment reduced LPS-induced pro-inflammatory phosphorylation; two patients presented reversal of immunosuppression after protracted exposure.

### 3.3. Risk of Bias

The risk of bias for the randomized trials included in the review was evaluated using the Cochrane RoB-2 tool by two independent reviewers, with a third reviewer resolving any disagreements. Johnson et al. (2025) [25] were judged to present some concerns, with low risk across the domains of randomization, deviations from intended interventions, missing outcome data, and outcome measurement, but with concerns related to selective reporting of results. Mastrofini et al. (2024) [26] were assessed as low risk across all domains. Morissette et al. (2021) [6] were determined to have high risk of bias, driven primarily by issues in the selection of the reported result. Urlic et al. (2023) [29] were rated as low risk across the evaluated domains.

Cohen et al. (2023) [20] demonstrated high risk, with concerns arising from the randomization process and selective reporting. Flores et al. (2023) [21] were categorized as some concerns, primarily in the domain of selection of the reported results. Gao et al. (2021) [22] were considered high risk, with notable issues in deviations from intended itnerventions. Gurgenci et al. (2023) [23] showed some concerns, particularly related to randomization and selection of the reported result.

**Figure 2:**
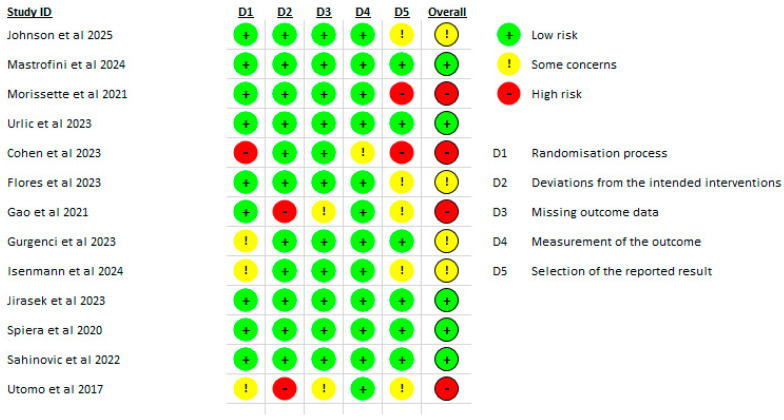
Bias assessment [6,17,20,21,22,23,24,25,26,27,28,29,30].

Isenmann et al. (2024) [17] were classified as some concerns, largely stemming from randomization and selection of the reported results. Jirasek et al. (2023) [24] were assessed as low risk in all domains. Spiera et al. (2020) [28] and Sahinovic et al. (2022) [27] were both rated as low risk, with no meaningful concerns identified. Finally, Utomo et al. (2017) [30] were determined to have high risk of bias, due to issues in deviations from intended interventions (Figure 2).

### 3.4. Synthesis of Results (Meta-Analyses)

**Figure 3:**
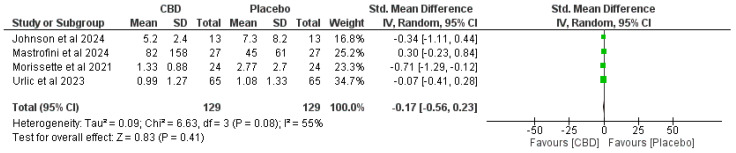
Forest plots comparing CBD to placebo for IL-6 [6,25,26,29].

**Figure 4:**
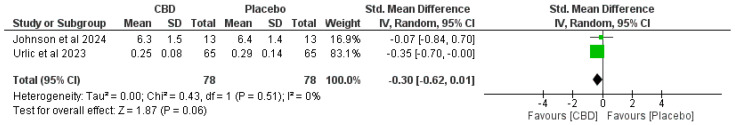
Forest plots comparing CBD to placebo for IL-8 [25,29].

**Figure 5:**
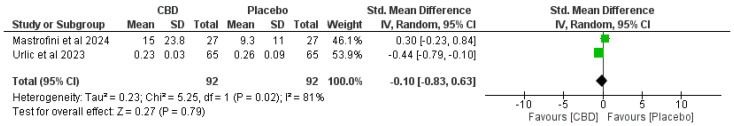
Forest plots comparing CBD to placebo for IL-10 [26,29].

**Figure 6:**
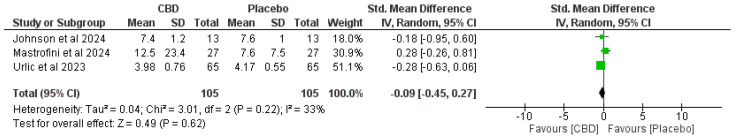
**TNF-α.** Forest plots comparing CBD to placebo for IL-10 [25,26,29].

Figure 3, Figure 4, Figure 5 and Figure 6 present forest plots comparing CBD to placebo for IL-6, IL-8, IL-10, and TNF-α.

1. **IL-6.** Four studies (total n = 129 per arm)—SMD −0.17 (95% CI −0.56 to 0.23), favoring CBD but not statistically significant (*p* = 0.41). Heterogeneity: I^2^ = 55% (Chi^2^ = 6.63, *p* = 0.08) (Figure 3).
2. **IL-8.** Two studies (n = 78 per arm)—SMD −0.30 (95% CI −0.62 to 0.01), trend preferring CBD but not statistically significant (*p* = 0.06). Heterogeneity: I^2^ = 0% (Chi^2^ = 0.43, *p* = 0.51) (Figure 3 and Figure 4).
3. **IL-10.** Two studies (n = 92 per arm)—SMD −0.10 (95% CI −0.83 to 0.63), no significant effect (*p* = 0.79). Heterogeneity: I^2^ = 81% (Chi^2^ = 5.25, *p* = 0.02) (Figure 5).
4. **TNF-α.** Three studies (n = 105 per arm)—SMD −0.09 (95% CI −0.45 to 0.27), no significant effect (*p* = 0.62). Heterogeneity: I^2^ = 33% (Chi^2^ = 3.01, *p* = 0.22) (Figure 6).

### 3.5. GRADE Assessment

Table 3 presents the bias assessment. Using GRADE, the certainty of evidence was regarded as follows:

1. **IL-6:** Very low certainty—downgraded for risk of bias, inconsistency, and imprecision.
2. **IL-8:** Moderate certainty—demoted one level for imprecision. No serious concerns for risk of bias, inconsistency, or indirectness.
3. **IL-10:** Low certainty—lowered for inconsistency and imprecision.
4. **TNF-α:** Moderate certainty—reduced one level for imprecision. No significant concerns about risk of bias, irregularity, or indirectness.

## 4. Discussion

Our synthesis reveals that phytocannabinoids (primarily cannabidiol (CBD) and, to a lesser extent, Δ9-tetrahydrocannabinol (THC)) yield inconsistent but biologically plausible effects on inflammatory biomarkers across clinical studies. Mechanistic preclinical literature delivers clear pathways, most notably inflammasome inhibition, NF-κB/MAPK modulation, and receptor-mediated immune reprogramming. Thus, it can elucidate the selective cytokine changes detected in some trials; however, conversion to strong, reproducible serum biomarker effects in humans remains limited by heterogeneous exposure, formulation, and population characteristics.

### 4.1. Mechanistic Rationale and Translational Limits

Preclinical evidence suggests that NLRP3 inflammasome inhibition is at the core of phytocannabinoid anti-inflammatory activity. Specifically, CBD and related cannabinoids reduce inflammasome assembly, caspase-1 activation, and downstream IL-1β/IL-18 maturation, while also attenuating NF-κB priming in multiple in vitro and animal models [31]. These upstream actions provide a mechanistic basis for the downstream reductions in IL-6, IL-8, and TNF-α reported intermittently in clinical studies. Still, most supporting data remain preclinical and vary widely by concentration, species, and cannabinoid type, so inflammasome-centered accounts must be applied carefully in clinical contexts [31].

Microglial and neuroimmune modulation further illustrate consistent mechanistic themes: cannabinoids repolarize microglia, inhibit NF-κB/MAPK and NLRP3 signaling, modulate endocannabinoid tone via FAAH, and preserve blood–brain barrier integrity, actions that credibly weaken local pro-inflammatory cascades [29]. Although this CNS-centered synthesis does not explicitly call for PK-embedded RCTs, it highlights the translational gap between mechanistic findings and human outcomes. It underscores the need for trials that directly link tissue exposure to biomarker responses [32].

Compound- and dose-specific complexities are evident in cellular studies. For instance, THCV, CBC, and CBN modulate the PANX-1/P2X7 axis and downregulate P-NF-κB and IL-6/TYK-2/STAT3 signaling in THP-1 macrophages, with dose-dependent, occasionally biphasic responses and inconsistent effects of CBN across assays [33]. Equally, BV2 microglial data reveal partial inhibition of NLRP3/caspase-1 by CBD, with mixed effects on ASC and TNF-α, indicating incomplete inflammasome blockade under acute pharmacologic exposure [12]. These results support the biological plausibility of the selective cytokine changes observed clinically (e.g., IL-1β). Still, they underscore significant translational limitations and the need for confirmation in primary human cells and in vivo PK/PD-matched studies [12,33].

### 4.2. Tissue Specificity and Route of Administration

A central theme across the scientific literature is tissue-specific pharmacology: local delivery (topical, oral mucosal, inhaled) often yields more robust tissue biomarker changes than systemic, low-bioavailability oral preparations. Reviews of cutaneous and periodontal cannabinoid pharmacology document epidermal and gingival ECS signaling (CB1/CB2, TRPV channels, PPARs), local antioxidant and anti-inflammatory effects, and antimicrobial/regenerative actions that can modify local biomarkers when suitable tissue exposure is attained [34,35]. These tissue-level mechanisms reasonably explain why topical or locally delivered cannabinoids produce pure local effects, while systemic serum cytokines remain mixed and often ineffective.

Clinical and trial evidence supporting route-dependent effects is persuasive. A 56-day RCT of a 1% CBD dental gel established significant reductions in gingival indices and trends toward reduced IL-6 and IL-8 [23]. Additionally, periodontal models demonstrate receptor upregulation and favorable shifts in bone-related signaling (RANK–RANKL–OPG) in response to phytocannabinoids, suggesting local tissue benefits that may not require systemic cytokine changes [34]. For cutaneous indications, multiple all-inclusive reviews and early clinical reports identify promising local signals for acne, atopic dermatitis, and seborrheic dermatitis. Still, they consistently underline physicochemical and formulation barriers to penetration and call for optimized delivery systems and local PK data [9,10,35].

### 4.3. Role of Advanced Formulations and Dermopharmacy

Nanoformulation and other dermopharmaceutical strategies substantially alter local exposure and effects: nanoparticulate carriers, nanoemulsions, microneedles, and cyclodextrin complexes increase dermal penetration, control cutaneous release, and can intensify local anti-inflammatory responses while limiting systemic absorption [8,10]. Ahmed et al.’s GRADE-based review ranks the evidence for enhanced skin penetration as high certainty. Still, clinical anti-inflammatory outcomes remain only moderately certain, as most supporting data are preclinical or from early-phase studies. Notably, some nanoformulations exhibit concentration-dependent cytotoxicity in vitro; thus, formulation-specific toxicology and larger RCTs are desirable before firm clinical adoption [8].

### 4.4. Systemic Exposure, Population Selection, and Dose

Systemic CBD exposure can curb soluble cytokines and immune cell phenotypes in humans under some conditions. In exploratory human trials—for instance, a high-dose CBD study in people with a cocaine use disorder—high systemic exposure was linked with reductions in IL-6 and VEGF-A and shifts in immune cell subsets, notwithstanding heterogeneity in plasma concentrations and sensitivity to analytical choices [6]. Similarly, recent healthy-adult trials and a sublingual hemp-derived CBD RCT failed to yield consistent changes in circulating TNF-α, IL-6, or IL-10 in unselected populations, and these trials often lacked serial PK sampling to assess exposure [26]. A crossover study using bioavailability-enhanced CBD (DehydraTECH) reported selective reductions in IL-8, IL-10, and IL-18 associated with blood pressure responses, but IL-1β and IL-6 decreased similarly after placebo, suggesting selective patterns and the need for replication and PK–PD linkage [35]. Similarly, these clinical data suggest that measurable immunomodulation is more likely to occur when systemic exposure is high, baseline inflammation is present, or the study population is pathophysiologically predisposed to immune dysregulation.

### 4.5. Limits of Current Clinical Trials and Implications for Interpretation

Several persistent methodological inadequacies limit confidence in clinical biomarker findings, including small sample sizes, underpowered secondary endpoints, heterogeneous formulations and doses, single-center designs, short durations, and the regular absence of serial pharmacokinetic measurements. For instance, a Parkinson’s disease trial with oral CBD provided no significant dermatologic benefit for seborrheic dermatitis but was underpowered for that secondary outcome and lacked robust PK–PD linkage [36]. Likewise, healthy-volunteer RCTs with single low-to-moderate daily doses and no serial PK sampling yielded null biomarker results that remain difficult to interpret relative to exposure [26]. These findings strongly support future trials that pair validated clinical endpoints with concurrent systemic and local PK sampling and pre-specified biomarker panels.

### 4.6. Heterogeneity Across Compounds and Assays

Heterogeneity arises not only from route and dose but also from the diversity of cannabinoid compounds and mixtures. Marijuana-derived phytocannabinoids can engage a CB2→PI3K axis that suppresses innate responses and exert antibacterial effects at concentrations that may similarly cause cytotoxicity in vitro [37]. Minor phytocannabinoids (THCV, CBC, CBN) show distinct and often opposing molecular effects on inflammasome-related pathways [33]. Such compound-specific and concentration-dependent differences mean that pooling across studies without accounting for formulation, exposure, and target tissue will obscure their true effects and inflate heterogeneity.

### 4.7. Practical Recommendations for Future Research

Founded on mechanistic and clinical evidence, we endorse the following:

1. Designing RCTs that include concurrent serial PK sampling (systemic and possibly local tissue or lesion sampling) to permit PK–PD linkage;
2. Prioritizing clinical populations with elevated baseline inflammation or condition-specific endpoints where local delivery is feasible;
3. Implementing dose-ranging and formulation-comparison arms to quantify exposure–response and safety;
4. Including mechanistic immune-cell phenotyping and pre-specified biomarker panels (including inflammasome markers, e.g., IL-1β/IL-18) to test hypothesized pathways; and finally;
5. Performing formulation-specific toxicology for advanced dermal/nanosystems in advance of large clinical trials [8,10,32].

## 5. Conclusions

Overall, preclinical and early clinical data converge on plausible immunomodulatory mechanisms for phytocannabinoids—particularly the inhibition of the NLRP3 inflammasome and suppression of NF-κB. Yet, clinical translation to consistent systemic biomarker changes is restricted by route, formulation, exposure, and population selection. Local (topical/oral mucosal) transfer and advanced formulation platforms reliably produce pronounced tissue effects that do not necessarily mirror systemic cytokine profiles; hence, topical and systemic evidence streams should be treated discretely and connected through careful PK–PD studies. Well-powered, PK-informed RCTs in selected clinical populations are mandatory before phytocannabinoids can be suggested as systemic anti-inflammatory agents.

## Figures and Tables

**Table 1:** Detailed search strings and databases.

Database	Search Strategy
PUBMED	(“Cannabidiol”[MeSH] OR “Epidiolex” OR “1,3-Benzenediol, 2-(3-methyl-6-(1-methylethenyl)-2-cyclohexen-1-yl)-5-pentyl-, (1R-trans)-” OR “Tetrahydrocannabinol”[MeSH] OR “Dronabinol”[MeSH] OR “THC” OR “Marinol”) AND (“Inflammation”[MeSH] OR “Cytokines”[MeSH] OR “Interleukins”[MeSH] OR “Inflammation” OR “Cytokine” OR “Interleukin”)
EMBASE	(‘cannabidiol’/exp OR ‘epidiolex’ OR ‘1,3-benzenediol, 2-(3-methyl-6-(1-methylethenyl)-2-cyclohexen-1-yl)-5-pentyl-, (1R-trans)-‘ OR ‘tetrahydrocannabinol’/exp OR ‘dronabinol’/exp OR ‘THC’ OR ‘marinol’) AND (‘inflammation’/exp OR ‘cytokine’/exp OR ‘interleukin’/exp OR ‘inflammation’ OR ‘cytokine’ OR ‘interleukin’)
LILACS	(“Cannabidiol” OR “Epidiolex” OR “1,3-Benzenediol, 2-(3-methyl-6-(1-methylethenyl)-2-cyclohexen-1-yl)-5-pentyl-, (1R-trans)-” OR “Tetrahydrocannabinol” OR “Dronabinol” OR “THC” OR “Marinol”) AND (“Inflammation” OR “Cytokine” OR “Interleukin”)
Cochrane Library	(“Cannabidiol” OR “Epidiolex” OR “1,3-Benzenediol, 2-(3-methyl-6-(1-methylethenyl)-2-cyclohexen-1-yl)-5-pentyl-, (1R-trans)-” OR “Tetrahydrocannabinol” OR “Dronabinol” OR “THC” OR “Marinol”) AND (“Inflammation” OR “Cytokine” OR “Interleukin”) IN Title, Abstract, Keywords
SCOPUS	TITLE-ABS-KEY(“Cannabidiol” OR “Epidiolex” OR “1,3-Benzenediol, 2-(3-methyl-6-(1-methylethenyl)-2-cyclohexen-1-yl)-5-pentyl-, (1R-trans)-” OR “Tetrahydrocannabinol” OR “Dronabinol” OR “THC” OR “Marinol”) AND TITLE-ABS-KEY(“Inflammation” OR “Cytokine” OR “Interleukin”)
Web of Science	TS=(“Cannabidiol” OR “Epidiolex” OR “1,3-Benzenediol, 2-(3-methyl-6-(1-methylethenyl)-2-cyclohexen-1-yl)-5-pentyl-, (1R-trans)-” OR “Tetrahydrocannabinol” OR “Dronabinol” OR “THC” OR “Marinol”) AND TS=(“Inflammation” OR “Cytokine” OR “Interleukin”)
CINAHL	(MH “Cannabidiol” OR “Epidiolex” OR “1,3-Benzenediol, 2-(3-methyl-6-(1-methylethenyl)-2-cyclohexen-1-yl)-5-pentyl-, (1R-trans)-” OR MH “Tetrahydrocannabinol” OR MH “Dronabinol” OR “THC” OR “Marinol”) AND (MH “Inflammation” OR MH “Cytokines” OR MH “Interleukins” OR “Inflammation” OR “Cytokine” OR “Interleukin”)

**Table 2:** Description of the characteristics of the study population of studies by author and year, sample, age (years), intervention, control, and outcomes.

Author/ Years	Study Design	Sample	Age (Years)	Intervention	Control	Outcomes
Cohen et al., 2023 [20]	Randomized, double-blind, placebo trial.	33 healthy participants.	Between 45 and 65 years old.	Administration: Topical cream containing 0.1% CBD (Echo Pharmaceuticals) and 0.1% EPA (fish oil-derived), plus 0.1% Salvia miltiorrhiza extract applied to the face. Dose: 1.17 ± 0.34 g/day. Duration: 56 days.	Identical formulation without the active ingredients.	Reduction in wrinkle volume and red spot count/area. Improved skin hydration (+31.2%), elasticity (+25.6%), and firmness after 56 days.
Flores et al., 2023 [21]	Double-blind, randomized, placebo-controlled trial.	Non-specified.	Between 18 and 50 years old.	CBD group (CG): CBD supplementation. Dose: 50 mg/day, after dinner, before sleep. Duration: 8 weeks.	Placebo group (PG): Placebo supplement. Dose: 225 mg/day, after dinner, before sleep. Duration: 8 weeks.	Inflammatory marker assessed: C-reactive protein (CRP, mg/L). Before intervention: CBD Group (CG): 1.5 ± 2 mg/L; Placebo Group (PG): 1.3 ± 1.6 mg/L. After intervention: CBD Group (CG): 1.3 ± 1.6 mg/L; Placebo Group (PG): 1.6 ± 2 mg/L. There were no significant differences between the groups in terms of CRP concentrations. Total range of CRP values in the study: 0.1 to 8.8 mg/L.
Gao et al., 2021 [22]	Interventional, double-blind, placebo-controlled study.	66 participants (57 completed the study) with atopic dermatitis involving >5% of the body surface (excluding the scalp).	Between 18 and 65 years old.	Group 1: JW-100 (pure hemp-derived CBD + aspartame). Group 2: Pure hemp-derived CBD. Group 3: Placebo. Administration: Topical application, at least twice a day, for 14 days.	Placebo group, which received a formulation without CBD.	The JW-100 group had the most significant reduction in ISGA score (1.28; *p* = 0.042) compared with placebo. In the subgroup of patients with an ISGA improvement of more than 2 points, 50% of the JW-100 group achieved “clear” or “almost clear” scores, compared with 15% in the placebo group (*p* = 0.028). No adverse events were reported in the treatment groups.
Gurgenci et al., 2023 [23]	Double-blind, randomized, placebo-controlled study. The MedCan-Infam study was a sub-study of MedCan-1.	MedCan-1: 141 participants with advanced cancer receiving palliative care. MedCan-Infam (sub-study): Convenience sample of 33 participants (15 in the CBD group, 17 in the placebo group).	Non-specified.	CBD oil, variable doses (50–600 mg/day), titrated according to tolerance. Administered orally. Duration: 28 days (dose escalation phase for the first 14 days).	Placebo (oil without CBD).	There was no significant difference in CRP (C-reactive protein) levels between the groups on day 14 or day 28. There was no difference in the trajectory of inflammatory cytokines between the CBD and placebo groups. The corticosteroids used by the patients did not significantly affect CRP levels.
Isenmann et al., 2024 [17]	Three-arm, double-blind, crossover study.	17 subjects (m = 15, f = 2).	Between 22 and 31 years old.	Administration: Oral. Substances used: CBD oil, CBD solution, and placebo. Dose: 60 mg/day. Duration: 6 consecutive days, followed by a washout period of at least 4 weeks between intervention cycles.	Each individual underwent the six-day high-intensity training protocol three times. After each training session, each individual received either a placebo or a CBD product (60 mg oil or 60 mg solubilized).	CBD oil use was associated with reduced myoglobin levels, a marker of muscle damage. This suggests that CBD may help minimize post-exercise muscle damage, promoting faster recovery. On the other hand, there were no significant differences in creatine kinase (CK) levels, another marker of muscle damage, indicating that CBD did not affect this aspect.
Jirasek et al., 2023 [24]	Placebo-controlled, double-blind, randomized study.	The research involved 90 participants, divided into three groups of 30 individuals each.	Between 18 and 65 years old.	Administration: Oral. Substances used: CBD (1% *w*/*w*), placebo (without CBD), and chlorhexidine digluconate 1% (active comparator group). Dose: Dental gel and toothpaste with 1% CBD or placebo, applied twice a day. Duration: 56 days.	Administration method: Toothpaste, as in the CBD groups. Composition: Did not contain cannabidiol (CBD). Appearance: Had the same appearance, flavor, and texture as products containing CBD, so that neither the participants nor the researchers knew who was using the active ingredient.	The use of 1% CBD toothpaste and gel for 56 days significantly reduced gingival inflammation. Participants who used the CBD products showed improvements in gingival indices and less bleeding than the placebo group.
Johnson et al., 2024 [25]	Randomized, double-blinded.	13 active males.	The average age of participants was 25 years.	Administration: Oral. Substances used: CBD and placebo. Dose: 298 mg of CBD. Duration: 105 min before exercise.	Ingested 298 mg CBD or placebo 105 min before 1 h treadmill exercise (60–65% VO_2_peak) in 32 °C and 50% relative humidity.	CBD caused a slight reduction in IL-6 concentration compared with placebo, suggesting a moderate decrease in the inflammatory response. In addition, CBD was associated with reduced monocyte activation, another inflammatory marker.
Mastrofini et al., 2024 [26]	Randomized, double-blind, placebo trial.	54 healthy males and females.	Between 18 and 32 years old.	Administration: Double-blinded, with both given in liquid form containing medium-chain triglyceride oil, while the CBD product specifically contained 50 mg/mL of CBD. Dose: Duration: 30 ± 3, 60 ± 3, and 90 ± 3.	Liquid was administered identically to the cannabidiol (CBD) product. Both products were presented in liquid form with medium-chain triglyceride oil and natural flavorings and were indistinguishable in appearance, taste, and smell.	Found no significant effects of cannabidiol (CBD) on inflammation in healthy adults. Concentrations of C-reactive protein (CRP), a standard marker of systemic inflammation, did not differ between the CBD and placebo groups throughout the study.
Morissette et al., 2021 [6]	Single-site randomized controlled trial.	48 participants. 83% of participants were men.	Between 18 and 65 years old.	Cannabidiol (CBD)—purified form (without THC), supplied by Tilray Inc. (New York, NY, USA) Administration: Oral, in gelatin capsules. Total daily dose of 800 mg, divided into two doses of 400 mg (morning and evening). 92 days (approximately 13 weeks) in total: Initial 10 days: Admission to a detox clinic. 12 weeks: Outpatient phase, with clinical monitoring and blood collections at 4 moments (baseline, day 8, week 4, and week 12).	Form: Gelatin capsules (same as those in the CBD group). Content: No active ingredient (no cannabidiol). Appearance and flavor: Identical to CBD capsules, to prevent participants from noticing any differences. Administration: Also in two daily doses (morning and evening), exactly imitating the protocol of the experimental group (800 mg/day in appearance).	CBD significantly reduced levels of interleukin-6 (IL-6) and VEGF-A, both of which are associated with chronic inflammation. In addition, it decreased the presence of pro-inflammatory immune cells, such as CD14^+^CD16^+^ monocytes and CD56^−^CD16^+^ NK cells, and increased the proportion of regulatory T cells (CD4^+^CD25^+^), which help control inflammation. These results suggest that CBD may modulate the immune system and attenuate the inflammatory response in this population.
Sahinovic et al., 2022 [27]	Randomized, double-blind design.	24 healthy adults participated in the study: 12 men and 12 women.	Between 18 and 35 years old.	Cannabidiol (CBD) isolate (no THC). Delivered in oral oil form. Oral, single dose, self-administered under researcher supervision 300 mg CBD. Single dose (acute, not chronic, administration study) CBD was administered 2 h before exercise to ensure adequate time for absorption and action.	Form: Oral oil, same as CBD. Content: the placebo oil did not contain cannabidiol but used the same oil base as the CBD formulation. Appearance: Identical to CBD (same volume and color), so participants did not know whether they were receiving CBD or a placebo.	CBD helped reduce muscle soreness and oxidative stress post-exercise. These results suggest that CBD may aid recovery and reduce inflammatory processes associated with intense physical exertion.
Spiera et al., 2020 [28]	Randomized, placebo-controlled trial.	42 subjects (27 in the lens group and 15 in the placebo group).	Between 18 and 69 years old; met the 2013 ACR/EULAR classification criteria for SSc.	Lenabasum-treated subjects received 5 mg once daily and placebo once daily; 20 mg once daily and placebo once daily; or 20 mg twice daily for the first 4 weeks. All subjects in the lenabasum group received 20 mg twice daily for the next 8 weeks.	Identical gelatin capsules in identical bottles, with similar labels and handling, but without the active ingredient.	Mean serum CRP, IL-6, and CXCL4 levels were comparable between treatment groups and within normal ranges. At baseline, CRP and IL-6 levels were each elevated in only 7 (17%) of 42 subjects.
Urlic et al., 2023 [29]	Randomized, double-blind, placebo-controlled, crossover study.	65 patients with Grade 1 and Grade 2 primary hypertension (as defined by the contemporary guidelines of the European Society of Cardiology for the treatment of hypertension).	Between 40 and 70 years old.	The dose of CBD ranged from 225 to 300 mg during the first 2.5 weeks, followed by an increase to 375–450 mg in the next 2.5 weeks, depending on participants’ sex and weight. After 5 weeks of dosing and a subsequent 2-week washout period, the participants who previously received CBD in the first 5 weeks were given a placebo for 5 weeks, and vice versa.	The placebo capsules were filled with an organic substrate powder ingredient devoid of any CBD active drug substance, as verified by HPLC testing, and were similarly filled into matching size 00 vegan gel capsules for blinding purposes.	5 weeks of oral CBD supplementation reduced serum levels of IL-8, IL-10, and IL-18. No significant dynamic in serum levels of PAI-1, LOX-1, and TNF-α was observed, whereas IL-1β and IL-6 reduced similarly after CBD and Placebo dosing.
Utomo et al., 2017 [30]	Randomized, double-blind, placebo-controlled, parallel design.	Patients suffering from chronic abdominal pain as a result of chronic pancreatitis or postsurgical pain.	18 years or older.	The add-on treatment consisted of two phases: a step-up phase (day 1–5: 3 mg three times a day (TID); day 6–10: 5 mg TID) and a stable dose phase (day 11–52: 8 mg TID). The dosage was tapered to at least 5 mg TID when 8 mg was not tolerated.	Identical step-up approach to the Namisol arm.	Downregulation of pro-inflammatory signaling, in particular that mediated by the stress-activated kinases p38MAP kinase and JNK, deactivation of the PKB/mTOR signaling cascade, and Ca^2+^ signaling are how THC drastically reduces S6 phosphorylation in CD3^+^ T cells.

**Legend:** CBD: Cannabidiol; SBP: Systolic blood pressure; DBP: Diastolic blood pressure; MBP: Mean blood pressure.

**Table 3:** Levels of evidence analysis via (GRADE Working Group, 2004).

Outcome	No. of Studies	Risk of Bias	Inconsistency	Indirectness	Imprecision	Certainty of Evidence
**IL-6**	4	Serious ^a^	Serious ^b^	Not serious	Serious ^c^	Very low
**IL-8**	2	Not serious	Not serious	Not serious	Serious ^d^	Moderate
**IL-10**	2	Not serious	Serious ^e^	Not serious	Serious ^f^	Low
**TNF-alpha**	2	Not serious	Not serious	Not serious	Serious ^g^	Moderate

Explanations: ^a^ Morissette et al. was judged “high risk” for selective reporting. ^b^ Moderate heterogeneity. I2 = 55%. ^c^ Pooled S MD = −0.17 (95% CI −0.56 to 0.23). ^d^ Pooled S MD = −0.30 (95% CI −0.62 to 0.01). ^e^ High heterogeneity. I2 = 81%. ^f^ Pooled S MD = −0.10 (95% CI −0.83 to 0.63). ^g^ Pooled S MD = −0.09 (95% CI −0.45 to 0.27).

## Data Availability

No new data were created or analyzed in this study. Data sharing is not applicable.

## Supplementary Materials

The data from this study are available at the following link (https://drive.google.com/drive/folders/1y90Lk4Iq3gkTxehDbVdwtU6WwMc98YfF?, accessed on 26 November 2025).

## Abbreviations

ACR/EULAR	American College of Rheumatology/European Alliance of Associations for Rheumatology
ASC	Apoptosis-associated speck-like protein containing a CARD
BBC	Blood–brain barrier
CBC	Cannabichromene
CBD	Cannabidiol
CBN	Cannabinol
CB1/CB2	Cannabinoid receptor type 1/type 2
CD4^+^, CD25^+^, CD14^+^, CD16^+^	Immune cell surface markers
CI	Confidence interval
CINAHL	Cumulative Index to Nursing and Allied Health Literature
CK	Creatine kinase
CRISS	Composite Response Index in Systemic Sclerosis
CRP	C-reactive protein
CXCL4	Chemokine (C-X-C motif) ligand 4
DBP	Diastolic blood pressure
EPA	Eicosapentaenoic acid
ERK	Extracellular signal-regulated kinase
FAAH	Fatty acid amide hydrolase
GRADE	Grading of Recommendations Assessment, Development, and Evaluation
HPLC	High-performance liquid chromatography
I-FABP	Intestinal fatty acid–binding protein
IL-1β	Interleukin-1 beta
IL-6	Interleukin-6
IL-8	Interleukin-8
IL-10	Interleukin-10
IL-18	Interleukin-18
ISGA	Investigator’s Static Global Assessment
I^2^	Inconsistency (heterogeneity) statistic
JNK	c-Jun N-terminal kinase
LILACS	Latin American and Caribbean Health Sciences Literature
LOX-1	Lectin-like oxidized low-density lipoprotein receptor-1
MAPK	Mitogen-activated protein kinase
MBP	Mean blood pressure
MCT	Medium-chain triglycerides
MD	Mean difference
MRSS	Modified Rodnan Skin Score
NLRP3	NOD-like receptor family pyrin domain containing 3
NK cells	Natural killer cells
NLM	National Library of Medicine
P2X7	Purinergic receptor P2X ligand-gated ion channel 7
PAI-1	Plasminogen activator inhibitor-1
PBMC	Peripheral blood mononuclear cells
PD	Pharmacodynamics
PGE2	Prostaglandin E2
PI3K	Phosphoinositide 3-kinase
PK	Pharmacokinetics
PICOS	Population, Intervention, Comparison, Outcomes, Study design
PKB/mTOR	Protein kinase B/mammalian target of rapamycin
PPAR-γ	Peroxisome proliferator-activated receptor gamma
PRISMA	Preferred Reporting Items for Systematic Reviews and Meta-Analyses
RCT	Randomized controlled trial
RERmax	Maximum respiratory exchange ratio
RevMan	Review Manager
RoB-2	Cochrane Risk of Bias tool version 2
SBP	Systolic blood pressure
SD	Standard deviation
SE	Standard error
SMD	Standardized mean difference
SSC	Systemic sclerosis
STAT3	Signal transducer and activator of transcription 3
TID	Three times a day
THC	Δ9-tetrahydrocannabinol
THCV	Tetrahydrocannabivarin
TNF-α	Tumor necrosis factor-alpha
TRPV	Transient receptor potential vanilloid
UVB	Ultraviolet B
VEGF-A	Vascular endothelial growth factor A
VO_2_max/VO_2_peak	Maximal/peak oxygen consumption
WHO ICTRP	World Health Organization International Clinical Trials Registry Platform

## References

[1] da Silva S.A.F., Guida H.L., Dos Santos Antonio A.M., de Abreu L.C., Monteiro C.B.M., Ferreira C., Ribeiro V.F., Barnabe V., Silva S.B., Fonseca F.L.A. (2014). Acute auditory stimulation with different styles of music influences cardiac autonomic regulation in men. Int. Cardiovasc. Res. J..

[2] Nascimento M.M.P., Azuelos A., Nóbrega I.L.P., Pitombeira M.S., Martinez A.T.A., de Carvalho J.F., Rodrigues C.E.M. (2025). State of the Art in Novel Treatment Strategies in Rheumatoid Arthritis: A Brief Review. Mediterr. J. Rheumatol..

[3] da Silva A.G., Guida H.L., Antônio A.M.d.S., Marcomini R.S., Fontes A.M., Carlos de Abreu L., Roque A.L., Silva S.B., Raimundo R.D., Ferreira C. (2014). An exploration of heart rate response to differing music rhythm and tempos. Complement. Ther. Clin. Pract..

[4] Meanti R., Bresciani E., Rizzi L., Molteni L., Coco S., Omeljaniuk R.J., Torsello A. (2025). Cannabinoid Receptor 2 (CB2R) as potential target for the pharmacological treatment of neurodegenerative diseases. Biomed. Pharmacother..

[5] Mujahid K., Rasheed M.S., Sabir A., Nam J., Ramzan T., Ashraf W., Imran I. (2025). Cannabidiol as an immune modulator: A comprehensive review. Saudi Pharm. J..

[6] Morissette F., Mongeau-Pérusse V., Rizkallah E., Thébault P., Lepage S., Brissette S., Bruneau J., Dubreucq S., Stip E., Cailhier J.F. (2021). Exploring cannabidiol effects on inflammatory markers in individuals with cocaine use disorder: A randomized controlled trial. Neuropsychopharmacology.

[7] Cásedas G., de Yarza-Sancho M., López V. (2024). Cannabidiol (CBD): A Systematic Review of Clinical and Preclinical Evidence in the Treatment of Pain. Pharmaceuticals.

[8] Ahmed B., Kaur S., Naryal S., Devi A., Kathpalia M., Shah R.M., Kaur I.P. (2025). Nanoformulated cannabidiol for skin disorders: A GRADE-based systematic review of therapeutic evidence and efficacy. Eur. J. Pharm. Biopharm..

[9] Kuzumi A., Yoshizaki-Ogawa A., Fukasawa T., Sato S., Yoshizaki A. (2024). The Potential Role of Cannabidiol in Cosmetic Dermatology: A Literature Review. Am. J. Clin. Dermatol..

[10] Rusu A., Farcaș A.M., Oancea O.L., Tanase C. (2025). Cannabidiol in Skin Health: A Comprehensive Review of Topical Applications in Dermatology and Cosmetic Science. Biomolecules.

[11] Sterne J.A.C., Savović J., Page M.J., Elbers R.G., Blencowe N.S., Boutron I., Cates C.J., Cheng H.Y., Corbett M.S., Eldridge S.M. (2019). RoB 2: A revised tool for assessing risk of bias in randomised trials. BMJ.

[12] Rodrigues F.D.S., Newton W.R., Tassinari I.D., da Cunha Xavier F.H., Marx A., de Fraga L.S., Wright K., Guedes R.P., Bambini-Jr V. (2024). Cannabidiol prevents LPS-induced inflammation by inhibiting the NLRP3 inflammasome and iNOS activity in BV2 microglia cells via CB2 receptors and PPARγ. Neurochem. Int..

[13] Martinez Naya N., Kelly J., Corna G., Golino M., Abbate A., Toldo S. (2023). Molecular and Cellular Mechanisms of Action of Cannabidiol. Molecules.

[14] Millar S.A., Stone N.L., Yates A.S., O’Sullivan S.E. (2018). A Systematic Review on the Pharmacokinetics of Cannabidiol in Humans. Front. Pharmacol..

[15] Hansen J.S., Boix F., Hasselstrøm J.B., Sørensen L.K., Kjolby M., Gustavsen S., Hansen R.M., Petersen T., Sellebjerg F., Kasch H. (2024). Pharmacokinetics and pharmacodynamics of cannabis-based medicine in a patient population included in a randomized, placebo-controlled, clinical trial. Clin. Transl. Sci..

[16] Yndart Arias A., Vadell K., Vashist A., Kolishetti N., Lakshmana M.K., Nair M., Liuzzi J.P. (2024). Cannabidiol, a plant-derived compound, is an emerging strategy for treating cognitive impairments: Comprehensive review of randomized trials. Front. Pharmacol..

[17] Isenmann E., Veit S., Flenker U., Lesch A., Lachenmeier D.W., Diel P. (2024). Influence of short-term chronic oral cannabidiol application on muscle recovery and performance after an intensive training protocol—A randomized double-blind crossover study. J. Int. Soc. Sports Nutr..

[18] Page M.J., McKenzie J.E., Bossuyt P.M., Boutron I., Hoffmann T.C., Mulrow C.D., Shamseer L., Tetzlaff J.M., Akl E.A., Brennan S.E. (2021). The PRISMA 2020 statement: An updated guideline for reporting systematic reviews. BMJ.

[19] Anil S.M., Peeri H., Koltai H. (2022). Medical Cannabis Activity Against Inflammation: Active Compounds and Modes of Action. Front. Pharmacol..

[20] Cohen G., Jakus J., Portillo M., Gvirtz R., Ogen-Shtern N., Silberstein E., Ayzenberg T., Rozenblat S. (2023). In vitro, ex vivo, and clinical evaluation of anti-aging gel containing EPA and CBD. J. Cosmet. Dermatol..

[21] Flores V.A., Kisiolek J.N., Ramani A., Townsend R., Rodriguez E., Butler B., Stewart L.K. (2023). Effects of Oral Cannabidiol on Health and Fitness in Healthy Adults: An 8-Week Randomized Trial. Nutrients.

[22] Gao Y., Li Y., Tan Y., Liu W., Ouaddi S., McCoy J., Kovacevic M., Situm M., Stanimirovic A., Li M. (2022). Novel cannabidiol aspartame combination treatment (JW-100) significantly reduces ISGA score in atopic dermatitis: Results from a randomized double-blinded placebo-controlled interventional study. J. Cosmet. Dermatol..

[23] Gurgenci T., Kijanka G., Greer R., Huggett G., Good P., Moniruzzaman M., Hardy J. (2023). Exploring potential anti-inflammatory effects of medicinal cannabis. Support. Care Cancer.

[24] Jirasek P., Jusku A., Frankova J., Urbankova M., Diabelko D., Ruzicka F., Papouskova B., Chytilova K., Vrba J., Havlasek J. (2024). Phytocannabinoids and gingival inflammation: Preclinical findings and a placebo-controlled double-blind randomized clinical trial with cannabidiol. J. Periodontal Res..

[25] Johnson D.A., Cable T.G., Funnell M.P., Peden D.L., Thorley J., Cunha M.F.D., Reynolds K.M., Harris L., Wood M., Chavez-O’Reilly T. (2025). Effects of Cannabidiol Ingestion on Thermoregulatory and Inflammatory Responses to Treadmill Exercise in the Heat in Recreationally Active Males. Med. Sci. Sports Exerc..

[26] Mastrofini G.F., McFadden B.A., Chandler A.J., Lints B.S., Cintineo H.P., Rhoades N.D., Vincenty C.S., Stray-Gundersen S.O., Lane A.D., Arent S.M. (2024). The effects of a brand-specific, hemp-derived cannabidiol product on physiological, biochemical, and psychometric outcomes in healthy adults: A double-blind, randomized clinical trial. J. Int. Soc. Sports Nutr..

[27] Sahinovic A., Irwin C., Doohan P.T., Kevin R.C., Cox A.J., Lau N.S., Desbrow B., Johnson N.A., Sabag A., Hislop M. (2022). Effects of Cannabidiol on Exercise Physiology and Bioenergetics: A Randomised Controlled Pilot Trial. Sports Med. Open.

[28] Spiera R., Hummers L., Chung L., Frech T.M., Domsic R., Hsu V., Furst D.E., Gordon J., Mayes M., Simms R. (2020). Safety and Efficacy of Lenabasum in a Phase II, Randomized, Placebo-Controlled Trial in Adults with Systemic Sclerosis. Arthritis Rheumatol..

[29] Urlic H., Kumric M., Dujic G., Vrdoljak J., Supe-Domic D., Dujic Z., Bozic J. (2023). Antihypertensive effects of CBD are mediated by altered inflammatory response: A sub-study of the HYPER-H21-4 trial. J. Funct. Foods.

[30] Utomo W.K., de Vries M., Braat H., Bruno M.J., Parikh K., Comalada M., Peppelenbosch M.P., van Goor H., Fuhler G.M. (2017). Modulation of Human Peripheral Blood Mononuclear Cell Signaling by Medicinal Cannabinoids. Front. Mol. Neurosci..

[31] Suryavanshi S.V., Kovalchuk I., Kovalchuk O. (2021). Cannabinoids as Key Regulators of Inflammasome Signaling: A Current Perspective. Front. Immunol..

[32] Tomaszewska-Zaremba D., Gajewska A., Misztal T. (2025). Anti-Inflammatory Effects of Cannabinoids in Therapy of Neurodegenerative Disorders and Inflammatory Diseases of the CNS. Int. J. Mol. Sci..

[33] Gojani E.G., Wang B., Li D.P., Kovalchuk O., Kovalchuk I. (2023). Anti-Inflammatory Effects of Minor Cannabinoids CBC, THCV, and CBN in Human Macrophages. Molecules.

[34] Carmona Rendón Y., Garzón H.S., Bueno-Silva B., Arce R.M., Suárez L.J. (2023). Cannabinoids in Periodontology: Where Are We Now?. Antibiotics.

[35] Ferreira B.P., Costa G., Mascarenhas-Melo F., Pires P.C., Heidarizadeh F., Giram P.S., Mazzola P.G., Cabral C., Veiga F., Paiva-Santos A.C. (2023). Skin applications of cannabidiol: Sources, effects, delivery systems, marketed formulations and safety. Phytochem. Rev..

[36] Weber I., Zagona-Prizio C., Sivesind T.E., Adelman M., Szeto M.D., Liu Y., Sillau S.H., Bainbridge J., Klawitter J., Sempio C. (2024). Oral Cannabidiol for Seborrheic Dermatitis in Patients with Parkinson Disease: Randomized Clinical Trial. JMIR Dermatol..

[37] Gu Z., Singh S., Niyogi R.G., Lamont G.J., Wang H., Lamont R.J., Scott D.A. (2019). Marijuana-Derived Cannabinoids Trigger a CB_2_/PI_3_K Axis of Suppression of the Innate Response to Oral Pathogens. Front. Immunol..

